# Air-Coupled Excitation of a Slow A_0_ Mode Wave in Thin Plastic Films by an Ultrasonic PMN-32%PT Array

**DOI:** 10.3390/s18093156

**Published:** 2018-09-19

**Authors:** Rymantas J. Kazys, Liudas Mazeika, Reimondas Sliteris, Justina Sestoke

**Affiliations:** Ultrasound Institute, Kaunas University of Technology, LT-51423 Kaunas, Lithuania; liudas.mazeika@ktu.lt (L.M.); reimondas.sliteris@ktu.lt (R.S.); justina.sestoke@ktu.lt (J.S.)

**Keywords:** air-coupled ultrasonic arrays, PMN-32%PT crystals, guided waves, non-destructive testing

## Abstract

Ultrasonic non-destructive testing techniques (NDT) based on the application of guided waves are already used for inspection of plate-type structures made of various materials, including composite materials. Air-coupled ultrasonic techniques are used to test such structures by means of guided waves. The objective of this research was development and investigation of air-coupled excitation of a slow A_0_ Lamb wave mode in thin plastic films by a PMN-32%PT ultrasonic array. It is known that when the velocity of the A_0_ mode in the film is less than the ultrasound velocity in air no leaky wave is observed in a surrounding air. It opens new possibilities for NDT of composite structures. The influence of the airborne wave may be eliminated by 3D filtering in a wavenumbers-frequency domain. A special filter and corresponding signals processing technique were developed in order to obtain directivity patterns and velocity maps of the waves propagating in all directions. The measured ultrasound velocity values prove that, with the proposed method, it is possible to excite a slow A_0_ Lamb wave mode and to separate it from other parasitic waves propagating in air. Measurements of the parameters of the slow A_0_ mode, such as the propagation velocity in the plastic film, may be applied for the material characterization.

## 1. Introduction

Ultrasonic guided waves are already widely used for non-destructive evaluation, structural health monitoring, and material characterization. They propagate in objects and structures in at least one dimension of which is comparable with the wavelength of the ultrasonic wave-pipes, rails, ropes, fuel tanks, and composite plates [1,2,3,4,5,6,7,8]. They allow increasing inspection speed and to inspect parts to which there is no direct access.

There are cases when guided ultrasonic waves may be excited only by contactless, e.g., air-coupled, techniques: hygroscopic materials like paper, thin flexible materials like plastic films, or specimens moving with a high velocity [9,10,11,12,13,14,15]. For non-destructive testing purposes, the antisymmetric A_0_ Lamb wave mode is widely used because it is sensitive to various types of defects, which are met especially in composite materials [10]. This mode is dispersive and its phase velocity is decreasing with a decreasing frequency. When the velocity of the A_0_ mode is higher than the ultrasound velocity in air a leaky wave is radiated into surrounding air. A different situation takes place when the velocity of the A_0_ mode becomes slower than the ultrasound velocity in air. Propagation of such waves is not accompanied by leaky waves into the surrounding air; therefore, there should be no losses due to this phenomenon [16]. Further, we shall call such a wave a slow Lamb wave A_0_ mode [17].

In spite of the fact that such a wave may be useful for non-destructive evaluation, until now there are not many publications dealing with air-coupled excitation and the detailed investigation of a slow A_0_ Lamb wave mode. The main problem, in this case, is that for an air-coupled excitation usually method applied by exploiting an ultrasonic transducer directed to the sample at the angle given by Snell’s law is not suitable, because such an angle does not exist. In order to overcome this problem we have proposed to use a planar multi-element array, which is placed close to the plate or film in which the required mode is excited [18]. However, this type of excitation has not been yet applied for the excitation of a slow A_0_ mode. Excitation via an air gap is usually accompanied by significant losses due to the mismatch of acoustic impedances of the ultrasonic transducer and air. In order to enhance the efficiency of the excitation of the A_0_ mode an ultrasonic array with PMN-32%PT crystals may be used. The good performance of such an array is obtained due to a very high electromechanical coupling coefficient *k* which, for the transverse extension mode, is *k*_32_ = (0.84–0.90) [19].

Air-coupled excitation of guided waves is accompanied by a rather strong ultrasonic wave propagating in air. Application of hardware shielding usually does not enable to separate signals created by a slow A_0_ mode and a wave in the surrounding air. For this purpose we propose to apply signal processing based on filtering in the frequency-wavenumber domains [20]. In our case, differently from the case analyzed in [21], the waves are propagating in all directions, which requires more complicated 3D filters to be applied. For a detailed material characterization we proposed specific filters in the frequency and spatial domains which enable the extraction of such properties as directivity patterns and velocity maps of the guided waves propagating in all directions of the planar structure under investigation.

The objective of the performed research was the development and investigation of air-coupled excitation of a slow A_0_ Lamb wave mode in thin plastic films by a PMN-32%PT ultrasonic array. The paper is organized as follows: The principle of excitation of the slow A_0_ mode by the air-coupled array and experimental investigations performed with a Polytec laser interferometer are presented in Section 2, Section 3 and Section 4. Theoretical analysis is based on the calculation of ultrasonic fields radiated by such an array and simulation of ultrasonic fields inside a thin plastic film. Problems arising in the case of low frequencies and correspondingly long wavelengths are discussed in Section 5. The calculation of ultrasonic fields in the plastic film is presented in Section 6. A signal processing method enabling the separation of the slow A_0_ mode in the plastic film from the influence of the wave propagating in air and to obtain parameters of the propagating A_0_ mode wave is described in Section 7. Discussion of the obtained results is presented in Section 8.

## 2. Excitation Principle

**Figure 1:**
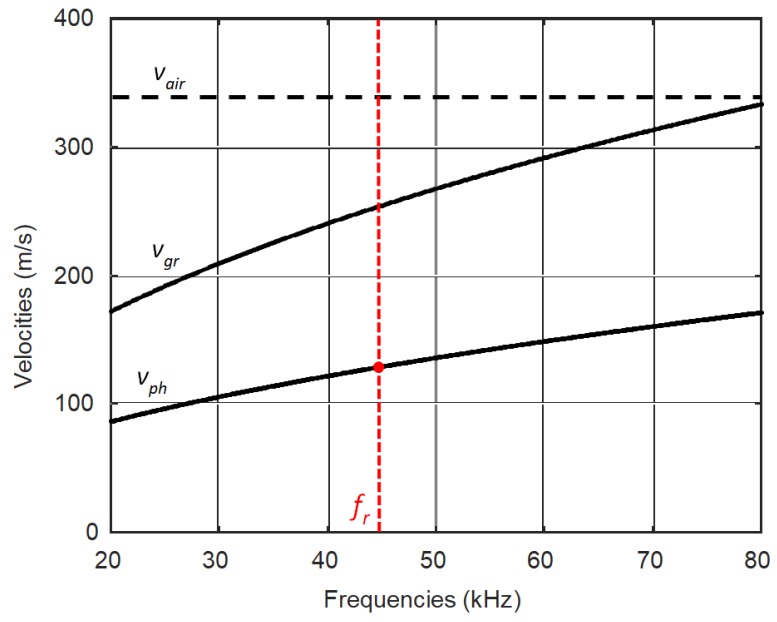
Dispersion curves of ultrasonic waves in a PVC film of 0.135 mm thickness: *v_ph_*—phase velocity of A_0_ mode, *v_gr_*—group velocity of the A_0_ mode, *v_air_*—velocity in air.

Air-coupled excitation of ultrasonic guided waves in plates is usually performed by ultrasonic transducers oriented to the surface of the plate at the angle $a_{opt}$ which is found from Snell‘s law:

(1) $$\;a_{opt}=arcsin\frac{v_{air}}{v_{ph}}\;$$

where *v_air_* air is the sound velocity in air, and *v_ph_* is the Lamb wave phase velocity. From Snell’s law it follows that air-coupled excitation is not feasible when *v_ph_* ≤ *v_air_* because such an angle, in this case the required deflection angle $a_{opt}$, should be more than 90°, does not exist. In thin plastic films and frequencies lower that 100 kHz the velocity of the antisymmetric A_0_ Lamb wave mode may be significantly lower that the ultrasound velocity in air, *v_air_* = 343 m/s. This is illustrated by the dispersion curves of the A_0_ mode presented in Figure 1.

Those curves were calculated by the semi-analytic finite element method [22]. The calculations were performed for polyvinyl chloride (PVC, London, UK) 0.135 mm thickness film, the main parameters of which are given in Table 1.

**Figure 2:**
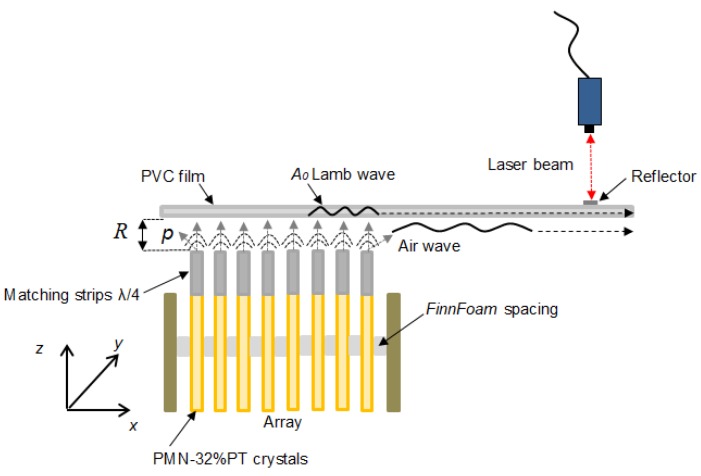
Excitation principle of ultrasonic guided wave by an air-coupled ultrasonic array.

From the results presented it follows that in the given frequency range up to 80 kHz the phase velocity of the A_0_ mode is much slower that the ultrasound velocity in air. In order to excite a slow A_0_ mode we propose to use a planar linear ultrasonic phased array consisting of strip-like piezoelectric elements with a rectangular aperture placed close to the film (Figure 2) [18,23]. Each piezoelectric element radiates an ultrasonic wave, which propagates via the air gap and excites a guided wave in the plastic film.

By selecting the operation frequency and adjusting delays of the excitation signals of the array elements, it is possible to excite efficiently an antisymmetric A_0_ mode Lamb wave in the film. The normal displacements caused by this mode were recorded by the laser interferometer. In order to obtain a suitable signal to noise ratio of the laser interferometer signals a very thin and very small reflecting film was glued to the film under investigation.

## 3. Air-Coupled PMN-32%PT Ultrasonic Array

In order to get a good efficiency of excitation as air-coupled array elements we have proposed to use PMN-32%PT piezoelectric crystals (HC Materials Corporation, Bolingbrook, IL, USA). The detailed analysis of the air-coupled array made of such elements may be found in [19]. However, to the best of our knowledge, PMN-32%PT piezoelectric elements have not yet been applied in ultrasonic arrays intended for air-coupled excitation of guided waves.

**Figure 3:**
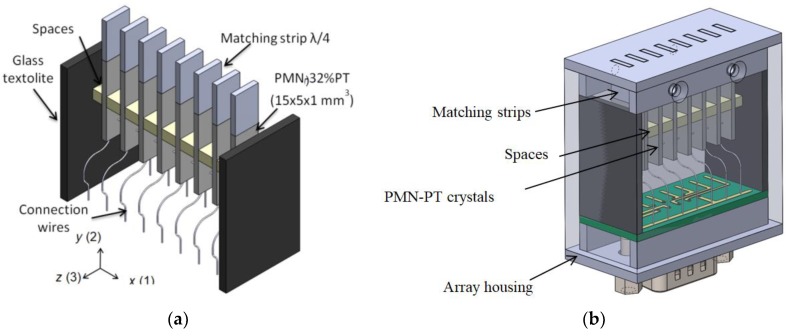
Air-coupled ultrasonic array: (**a**) with the matching strips; and (**b**) transparent view.

The ultrasonic array consisting of eight PMN-32%PT crystal strips 15 × 5 × 1 mm was manufactured (Figure 3). For radiation, the edge perpendicular to the *z* axis was selected. The strip-like piezoelectric elements vibrate in the transverse extension mode for which the electromechanical coupling coefficient *k*_32_ = 0.84–0.90. For improvement of the performance special acoustic matching elements of a low acoustic impedance (0.268 MRayl) plastic strips made of AIREX T90.210 type polystyrene foam (AIREX AG, Sins, Switzerland) were bonded to the active edge of the crystal [19]. The length of the matching strips is λ/4 at the operation frequency. The array elements are separated by a spacing of 2 mm, which are made of a low-density (ρ = 38 kg/m^3^) *FinnFoam* material (Finnfoam OY, Salo, Finland), with the width and the height of 5 mm and 3 mm, correspondingly. They enable minimizing the acoustic cross-talks between piezoelectric elements in the array.

For efficient excitation of the A_0_ mode, the lateral dimensions of the strip-like elements are selected less than the half wavelength. The pitch between the elements is approximately equal to the wavelength of the A_0_ mode in the film. For the analyzed case, this wavelength at the frequency 40 kHz is 3.2 mm. Therefore, the dimensions of the radiating aperture of the individual array elements were selected 1 × 5 mm. For excitation of the A_0_ mode, the array elements are excited successively with the time delay necessary for this mode to propagate the distance between the adjacent elements:

(2) $$\Delta \tau_{d}=\frac{\lambda_{A_{0}}}{v_{ph}\left(f,h\right)}$$

where λ_A0_ is the wavelength of the A_0_ mode, *h* is the thickness of the PVC film, and *f* is the frequency. In this case, each array element excites the A_0_ mode in phase, thus increasing the amplitude of the propagating guided wave.

Please note that the velocity of the A_0_ mode is frequency-dependent; therefore, the pitch between the array elements should depend on the operation frequency *f* also. However, it is necessary to point out that instead of changing the pitch the same result may be obtained by introducing delays between excitation instants of the array elements in addition to the delays given by Equation (2). The obtained result will be discussed below.

## 4. Measurements by the Laser Interferometer

**Figure 4:**
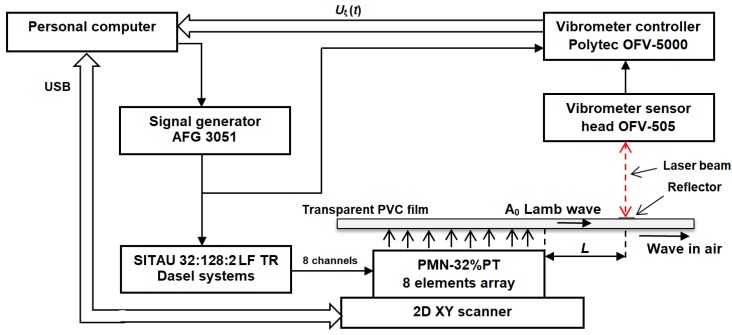
Experimental setup.

In order to estimate the operation of the air-coupled array and to measure normal displacements of the excited guided waves in a thin plate the laser interferometer method was applied. The experimental setup used for this purpose is shown in Figure 4. This setup consists of the laser interferometer (OFV-5000 and sensor head OFV-505, Polytec GmbH, Waldbronn, Germany), an AFG 3051 signal generator (GW INSTEK, UK), a Dasel SITAU 32:128:2 LF TR multi-channel system (Dasel sistemas, Madrid, Spain), a 2D scanner (Standa, Vilnius, Lithuania), the ultrasonic air-coupled array, a personal computer, and the transparent polyvinyl chloride (PVC) film fixed on a bracket. For experiments, a thin film 210 × 297 mm of 0.135 mm thickness was selected. The phased array was positioned in the center of the film along all axes. The PVC film vas fixed on a rectangular frame at the edges of the film without any additional tension.

In most cases, the air-coupled linear eight-element array was excited by a 43 kHz and three-period electric burst. The spatial distribution of normal displacements was measured by scanning the air-coupled array in the *x* and *y* directions with 0.1–0.5 mm scanning steps. The array was placed close to the film. In different experiments, the distance was set in the range of 0.1–1 mm.

**Figure 5:**
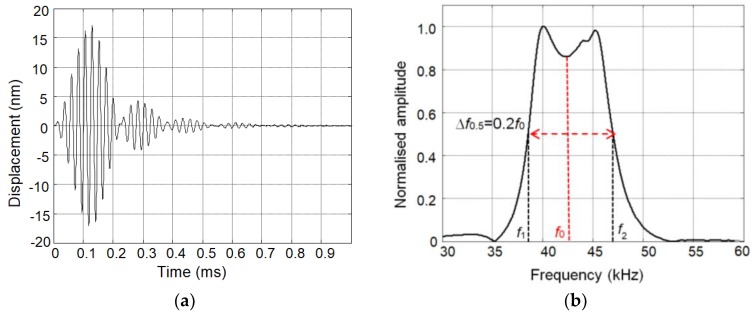
The measured ultrasonic signals of single element of the air-coupled array: (**a**) waveform; and (**b**) normalized spectrum.

The waveform of the ultrasonic pulse on the tip of the individual array element with the matching strip recorded by the laser interferometer is shown in Figure 5a. In Figure 5b is shown the spectrum of this signal. The element was excited by the electric pulse with the amplitude 50 mV, the duration of three periods, and a central frequency of 43 kHz. The presented results show a wide bandwidth (∆*f* = 0.2 *f*_0_, where *f*_0_ is the central frequency) and a good performance in the time domain.

**Figure 6:**
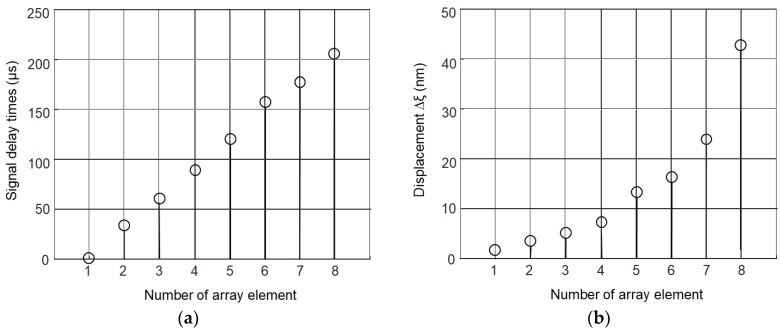
Optimization of excitation of the air-coupled ultrasonic array: (**a**) delay times of the excitation instants of different array elements; and (**b**) normal displacements of the PVC film versus the number of excited array elements.

Such pulses with the appropriate delays given by Equation (2) were used to excite the A_0_ mode in the PVC film. In order to optimize the performance of the array from the point of a view of excitation of the A_0_ mode the measurements of normal displacements of the film were performed at the distance *L* = 1 mm from the eighth element of the array on the right side. Excitation of the array elements was performed by a Dasel SITAU 32:128:2 LF TR multi-channel system. The time delays between excitation signals of the array elements were changed with respect to the delays given by Equation (2) until the maximal amplitude of the normal displacement of the film at the distance *L* = 1 mm from the eighth element was obtained. The delay times obtained in such way are depicted in Figure 6a. Figure 6b shows how the amplitude of the excited normal displacement of the film grows with the increasing number of the excited array elements. This improvement is significant—from 1.6 nm in the one element case to 43 nm in the of the whole eight-element array.

The experiments show possibility to excite with the proposed air-coupled array a slow ultrasonic A_0_ wave in plates, e.g., when the phase velocity of those waves is slower than the ultrasound velocity in air. However, the experiments revealed problems caused by a low frequency and correspondingly long wavelength of ultrasonic signals.

The distance between the array and the film was rather short—1 mm. In spite of such a short distance the radiated ultrasonic wave due to a low frequency and a correspondingly long wavelength in air which, at 43 kHz is λ_A_ = 8.55 mm, is spreading and covering a wider zone than the aperture of the individual elements of the array. This phenomenon may significantly reduce the efficiency of the excitation of the slow A_0_ mode in the film. Let us analyze this problem in detail.

## 5. Calculation of the Ultrasonic Field in the Air Gap

Acoustic fields radiated by the array consisting of elements with a rectangular aperture were calculated using the impulse response method (IRM). The pressure field at a given point *P* is found from the impulse response *h*(*P,t*) and vibration velocity *v_i_*(*t*) of the radiating aperture:

(3) $$\;p\left(P,t\right)=\rho \frac{\partial v\left(t\right)}{\partial t}*h\left(p,t\right)\;$$

The symbol ∗ denotes a convolution, the expression of the impulse response *h*(*P,t*) for a rectangular aperture may be found elsewhere [24,25]. The acoustic field radiated by the whole array is obtained as a sum of fields *p_i_* radiated by individual elements:

(4) $$\;p\left(P,t\right)=\overset{N}{\underset{i=1}{\sum }}p_{i}\left(P,t\right)\;$$

where *N* is the number of elements in the air-coupled ultrasonic array. This means that for the calculation of the acoustic fields in air, vibration velocities *v_i_(t*) of all the radiating rectangular apertures are required. This is a natural extension of the method for predicting the pressure field from a rectangular source by using the impulse response method [26,27,28,29,30,31].

Calculations of the acoustic fields by this method were performed using an open source software tool *The Lamb Matlab Toolbox* presented in [32,33]. This software tool was adapted in terms of generation of the excitation signal, description of the array geometry, calculation of the coordinate points in excitation and radiation zones. A new set of functions for visualization of the acoustic pressure fields radiated by the air-coupled array was created.

**Figure 7:**
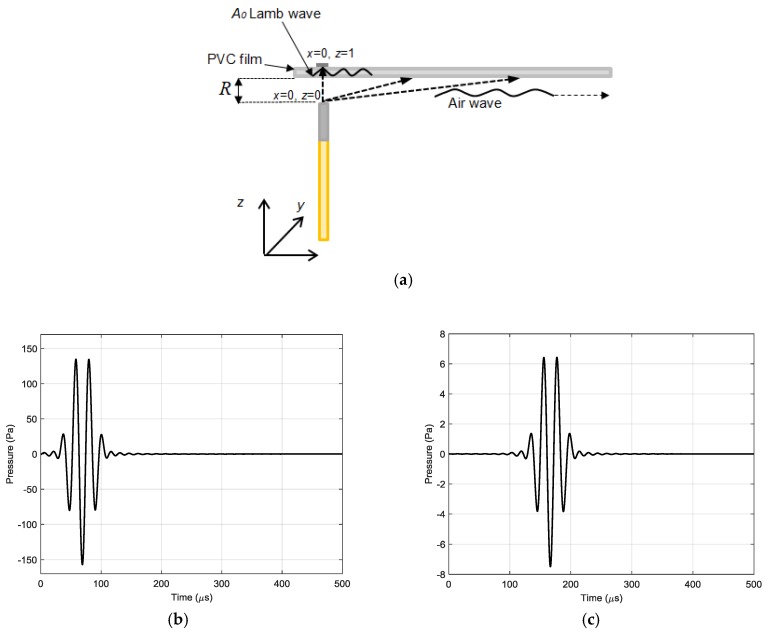
Calculated waveforms of the incident ultrasonic pressure pulses in the plane of the plastic film (z = 1 mm) at different distances from the piezo element symmetry axis: (**a**) schematic diagram showing propagation directions of ultrasonic pulses; (**b**) *x* = 0 mm (piezo element centre); and (**c**) *x* = 35 mm.

**Figure 8:**
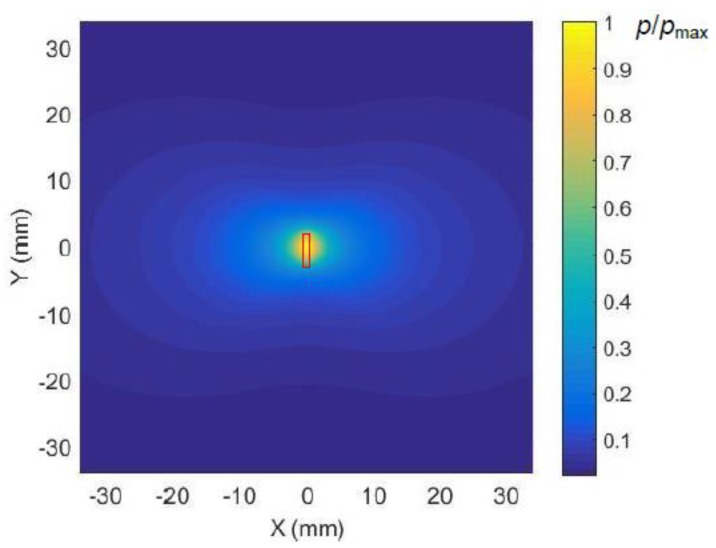
The calculated spatial distribution of maximal values of the ultrasonic pressure field in air radiated by a single rectangular array element (shown by a solid red line) field in the plane of the film at the distance *R* = 1 mm from the element. The presented amplitude values shown in the color bar were normalized with respect to the maximum value in the field.

The acoustic pressure fields radiated by the array were calculated in the plane coinciding with the plastic film at the distance *R* = 1 mm from the array. In order to evaluate the influence of ultrasonic field spreading due to diffraction the calculations first were performed for a single array element with a rectangular aperture with dimensions 1 × 5 mm. The waveforms of the ultrasonic pressure pulses at two different distances *x* = 0 mm and *x* = 35 mm are shown in Figure 7b,c. Please note a significantly lower amplitude of the pulse at the distance *x* = 35 mm. The 2D spatial distribution of the acoustic pressure field in the plane coinciding with the film, e.g., at the distance *R* = 1 mm from the array element, is presented in Figure 8.

**Figure 9:**
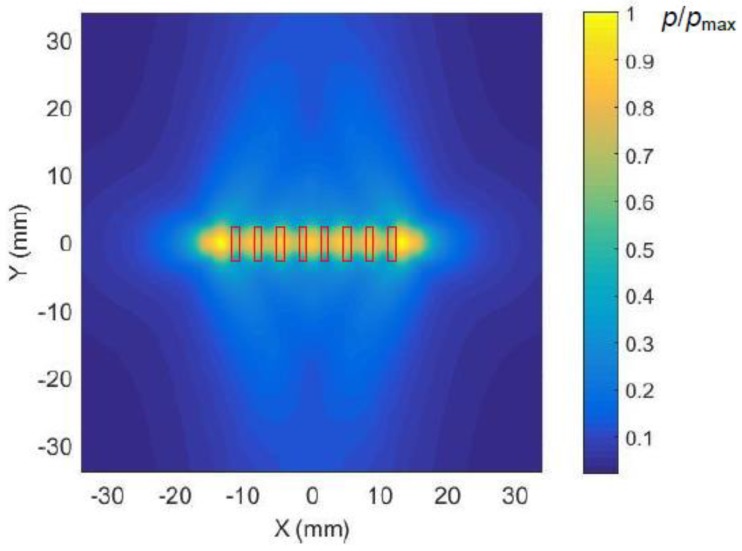
Calculated ultrasonic pressure field in air radiated by the eight-element rectangular array at the distance *R* = 1 mm from the array when all elements of the array are excited simultaneously. The presented amplitude values shown in the color bar were normalized with respect to the maximum value in the field.

**Figure 10:**
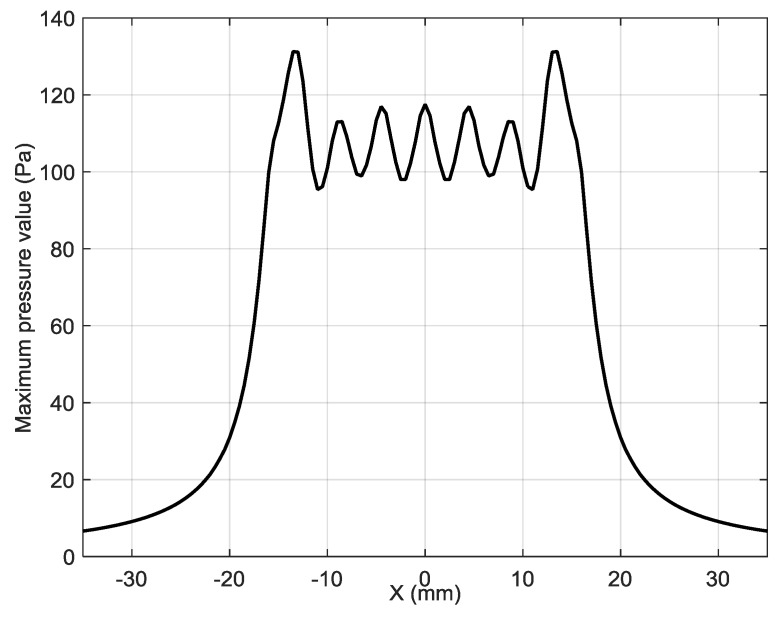
The spatial distribution of the acoustic pressure along the center line of the eight-element array in the plane of the film (*R* = 1 mm) when all elements of the array are excited simultaneously.

The ultrasonic pressure field radiated by the eight-element array when all elements of the array are excited simultaneously is shown in Figure 9. The corresponding spatial distribution of the acoustic pressure along *x* axis is given in Figure 10. This field is a result of interference of ultrasonic fields radiated by all array elements and is not efficient for excitation of the A_0_ mode.

From the results presented follows that the distribution of the acoustic pressure along *x* axis is obviously wider than the aperture of the individual array elements. It means that radiated via the air gap ultrasonic field will excite the guided wave not only in front of the particular array element, but also quite far away from it, however, with a time delay defined by the ultrasound velocity in air, but not by the A_0_ mode phase velocity in the film. As it was mentioned above, it may reduce the efficiency of the excitation of a guided A_0_ mode.

**Figure 11:**
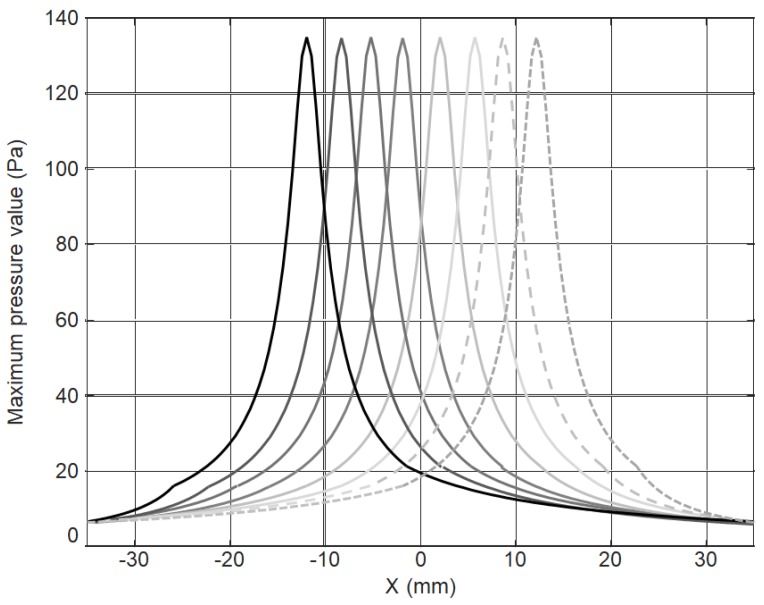
The spatial distribution of the acoustic pressure along the center line of the array at the distance *R* = 1 mm from the array at different time instants.

If to replace the simultaneous excitation of all array elements by a successive excitation element-by-element with the time delays given by Equation (2) then the obtained pressure fields in the successive time instants in the plane of the film along the center line of the array are shown in Figure 11 by different curves. In this case, the exciting pressure field is moving with the propagating A_0_ guided wave mode with the same velocity. Such fields are more suitable for excitation of the selected guided wave.

## 6. Calculation of the Ultrasonic Fields in the Plastic Film

In order to evaluate feasibility and efficiency of the excitation of a slow A_0_ mode at low frequencies first we shall analyze excitation of ultrasonic guided waves in the plastic film by a single array element with a rectangular aperture. Please note that in the analyzed case, the excitation zone is not limited by the aperture 1 × 5 mm, but as it follows from the calculated ultrasonic field in air (Figure 8) it is significantly wider than the width of the 1 mm element. It is necessary to point out that at the points located outside of the rectangular aperture of the array element (denoted in Figure 11 by the yellow lines) the exciting ultrasonic waves propagating through air arrive at any point in the plate earlier than the A_0_ mode just excited under the array element because the ultrasound velocity in air in the analyzed case is higher than of the slow A_0_ mode.

For calculation of the spatial distribution of normal displacements in the excited guided wave, we shall use the Huygens’s principle-each point on the surface of the film affected by the incident acoustic pressure becomes a source of cylindrically propagating waves. Transformation of the pressure *p* in air into normal displacement ξ of the film can be characterized by the transformation coefficient *k* = ξ/*p* which in our case was determined experimentally and is 18 nm/Pa. The incident pressure *p* with frequency 43 kHz was measured by a 1/8 inch Brüel and Kjær microphone (B&K 4138-A-015, Brüel and Kjær, Naerum, Denmark) at the distance *R* = 1 mm from the radiating aperture. The excited displacement field in the film was measured by a OFV-5000 Polytec laser interferometer (Polytec GmbH, Waldbronn, Germany). However, for analysis of a spatial structure of the excited normal displacement fields absolute values are not important, therefore, we shall use the displacement amplitudes normalized with respect to the maximum value of the analyzed field.

**Figure 12:**
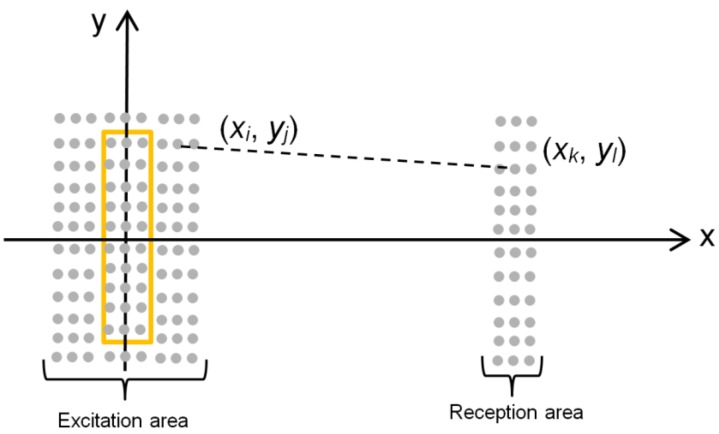
Excitation and reception areas.

For calculations in excitation and reception areas, a grid of points *x_i_*, *y_j_* is formed (Figure 12). The distance between adjacent points in our case was 0.5 mm. This means that across the radiating aperture there are three points at which the incident pressure waveform is defined. The wave from an arbitrary point *x_i_*, *y_j_* in the excitation area propagates uniformly in all directions and may be calculated at the selected point *x_k_*, *y_l_* in the reception area (Figure 12).

In this case, the guided wave propagates the distance:

(5) $$\;d_{ij,\;kl}=\sqrt{{\left(x_{k}-x_{i}\right)}^{2}+{\left(y_{l}-y_{j}\right)}^{2}}\;$$

Usually for measurements pulse type signals are used. Please note that the waveforms of the displacements at the different points in the excitation area are obtained from the calculation of the incident ultrasonic pressure field in the air gap presented in Section 4. Taking into account that an A_0_ mode is highly dispersive analysis must be performed for each spectral component. For this purpose, let us calculate the spectrum *S* (*f*, *x_i_*, *y_j_*) by the Fourier transform:

(6) $$\;S\left(f,\;x_{i},\;y_{j}\right)=FFT\left[\xi \left(t,x_{i},y_{j}\right)\right]\;$$

where FFT is the fast Fourier transform, *ξ*(*t*, *x_i_*, *y_j_*) is the displacement at the point *x_i_, y_j_* excited by the incident ultrasonic pressure wave. Then the spectrum of the displacement at the reception point *x_k_*, *y_l_* is obtained from:

(7) $$\;S\left(f,x_{k},y_{l},x_{i},y_{j}\right)=\frac{1}{\sqrt{d_{ij,kl}}}\cdot S\left(f,x_{i},y_{j}\right)\cdot H\left(f,d_{ij,kl},v_{ph}\right)\;$$

where *H*(*f*, *d_ij_*,*_kl_ v_ph_*) is the transfer function of the plastic film for the A_0_ mode [34]. The transfer function is given by:

(8) $$\;H\left(f,d_{ij,kl},v_{ph}\right)=e^{-\alpha \left(f\right)\cdot d_{ij,kl}}\cdot e^{-j^{\left(\frac{2\pi fd_{ij,kl}}{v_{ph}\left(f,h\right)}\right)}}\;$$

where α(*f*) is the frequency *f* dependent attenuation coefficient, *v_ph_* is the phase velocity of the A_0_ mode, and *h* is the thickness of the film.

The total displacement at the reception point *x_k_*, *y_l_* is formed by the particular waves arriving from all points taken into account in the excitation area:

(9) $$\;S_{T}\left(f,x_{k},y_{l}\right)=\overset{N}{\underset{i=1}{\sum }}\overset{M}{\underset{j=1}{\sum }}S\left(f,x_{k},y_{l},x_{i},y_{j}\right)\;$$

where *N* and *M* are the numbers of the points in the excitation area correspondingly in *x* and *y* directions.

The waveform at the reception point *x_k_*, *y_l_* is obtained as a real part of the inverse Fourier transform [35]:

(10) $$\;\xi \left(t,x_{k},y_{l}\right)=FFT^{-1}\left[S_{T}\left(f,x_{k},y_{l}\right)\right]\;$$

In order to get the whole displacement field of the plastic film in the designated reception area the calculation must be performed for all points *x_k_*, *k* = 1…*N* and *y_l_*, *l* = 1…*M*.

**Figure 13:**
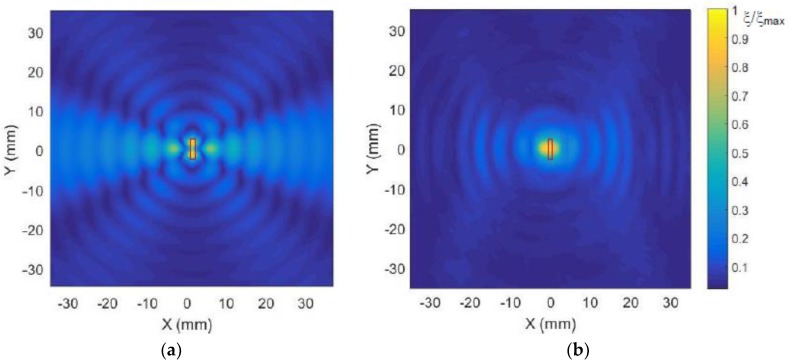
2D spatial distributions of normal displacements ξ of the plastic film taking into account interference of the direct A_0_ mode and additional wave caused by the wave propagating in air: (**a**) calculated; (**b**) measured. The presented amplitude values shown in the color bar were normalized with respect to the maximum value in the field.

The spatial distributions of normal displacements ξ in the film excited by a single element via an air gap with a thickness of 1 mm and calculated by the described method are shown in Figure 13a. The position and orientation of the single array element is denoted by the black rectangle. The displacement field measured by the laser interferometer is presented in Figure 13b. It is necessary to point out in the presented figure’s interference pattern of two waves—the A_0_ mode and wave propagating in air is shown. From the presented distributions also follows that, due to a rectangular aperture, the propagation of guided waves is not uniform in all directions. The most intense radiation is observed along the *x* axis, e.g., in the direction perpendicular the longest leg of the rectangular aperture. This means that such rectangular elements are suitable for the air-coupled array intended for excitation of guided waves.

**Figure 14:**
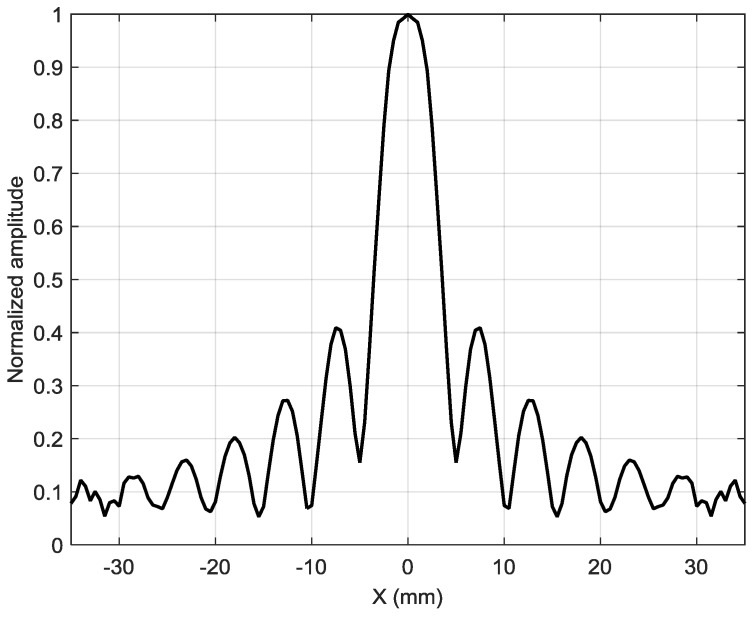
Calculated spatial distribution of the normalized displacement amplitudes in the plastic film along the *x* axis at y = 0 excited by a single rectangular element.

Spatial distribution of the calculated amplitude variations along the *x* axis is shown in Figure 14. Let us analyze how the array composed of such elements will operate.

On the other hand, it is possible to see periodic oscillations in the field which are caused by interference of ultrasonic guided waves arriving from different points in the excitation area and through air. Some difference between the simulation and experiment is due to different ratios of the amplitudes of the wave propagating in the film and air.

**Figure 15:**
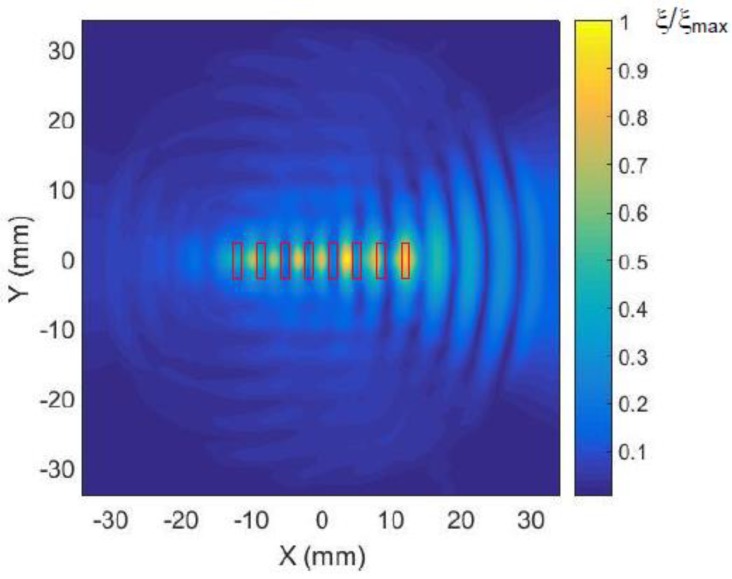
Calculated 2D spatial distribution of normal displacements ξ of the plastic film excited by the eight-element phased air-coupled array. The presented amplitude values shown in the color bar were normalized with respect to the maximum value in the field.

In Figure 15 the displacement field excited in the plastic film by the eight-element air-coupled array is presented. The array was excited in a phased mode-the array elements were excited by the signals, which were delayed according to the velocity of A_0_ mode propagating in the film. The waveform of the signal radiated by the array elements is shown in Figure 7b. The delays of the exciting signals were selected in the order to get radiation to the right side of the array. The used delay times are presented in Figure 6. From the results presented follows that the most intense radiation is, in fact, observed along *x* axis.

Please note that the observed amplitude variations in Figure 15 do not show only propagation of A_0_ mode in the film, but the result of the interference of the waves propagating in the film and air. Selecting only one type of wave is possible by spatial–temporal 3D filtering described in Section 7.

**Figure 16:**
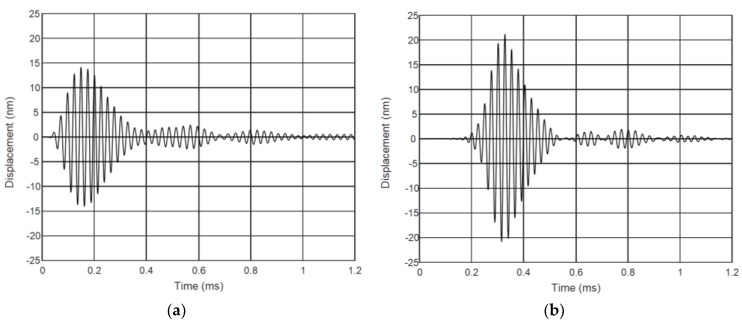
Measured normal displacements ξ of the plastic film excited by the 8 element air-coupled array at *L* = 1 mm from the last array element: (**a**) all array elements are excited simultaneously; and (**b**) successive (phased) excitation of the array elements with the optimized delay times.

The measured ultrasonic waveforms of normal displacements of the plastic film excited by the air-coupled array are presented in Figure 16. The mechanical displacements were measured near piezoelectric element No. 8 at a distance *L* = 1 mm from the element center.

**Figure 17:**
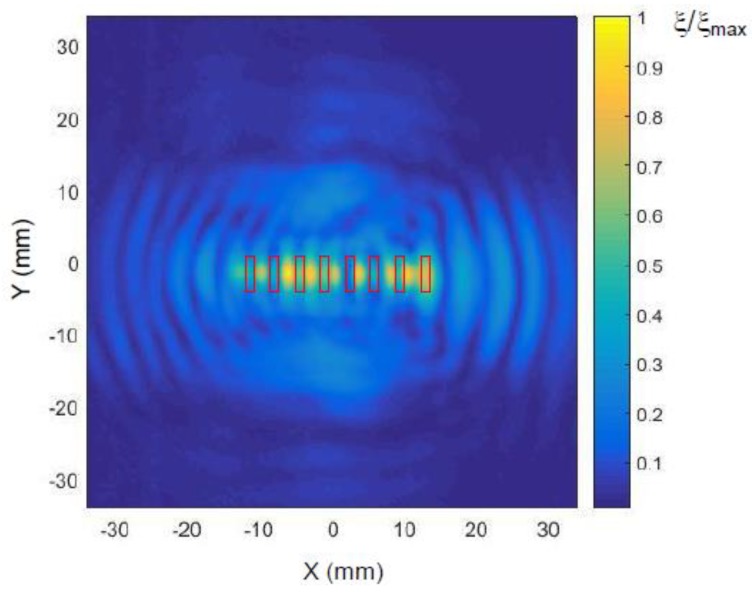
Measured 2D spatial distribution of normal displacements ξ of the plastic film excited by the eight-element phased air-coupled array. The presented amplitude values shown in the color bar were normalized with respect to the maximum value in the field.

From the results presented follows that the phased excitation of the array (Figure 16b) enables obtaining higher amplitude (almost 1.5 times) and the three-times lower tail of the ultrasonic impulse. The measured C-scan of the field excited in the plastic film with the phased array is shown in Figure 17.

**Figure 18:**
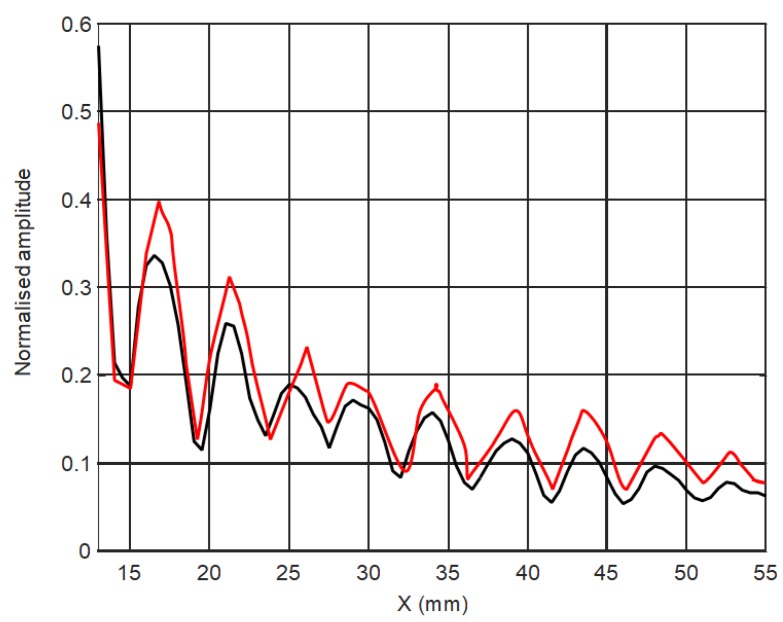
The spatial distribution of the normalized displacement amplitudes in the plastic film excited by the eight-element air-coupled array: black color—measured, red—calculated.

However, in this case strong amplitude oscillations along the *x* axis are also observed (Figure 18). As it was mentioned above, they are caused by interference of the waves propagating in the plastic film from different points of a rather wide excitation area and air.

In order to exploit such waves for NDT and material characterization only the wave propagating from well-defined small excitation area would be preferable, however, the performed experiments have shown that to achieve such objective with various hardware solutions (shielding of waves propagating in air, etc.) at used low frequencies (43 kHz) actually is impossible. Therefore, for efficient selection of necessary types of waves we have proposed to apply a signal processing method based on 3D filtering in spatial–time domains. This method and the obtained results are presented in Section 7.

## 7. Separation of Guided Waves by 3D Filtering

From the performed theoretical analysis and experiments follows that at each point of the flexible film normal displacements are caused by the guided A_0_ mode propagating in the film and by the wave propagating in air. Those waves are propagating with different velocities and their interference is producing the resultant normal displacements of the film. For material evaluation purposes the vibrations caused by the airborne wave act as a disturbing artefact. Their influence may be reduced by a signal processing of the collected B-scans of the normal displacements u(x,t) along some selected direction, for example, *x*. Usually such signal processing is based on a three-dimensional Fourier transform of the data set *u(x,y,t)* and two-dimensional filtering of the obtained spatial–temporal spectrum *U*(*f_x_, f_y_, f*), where *f_x_* and *f_y_* are the spatial frequencies. Such processing enables the determination of dispersion curves of different wave modes and, consequently, to separate wave modes propagating with different velocities [20,21]. In the presented works, the authors investigated waves propagating only in a positive half plane of the *x* axis. Thus, in order to separate necessary propagating modes the filtering was performed slice by slice separately for a corresponding cross-section of the 3D spectrum using a 2D filter. Such an approach takes a longer time as it requires many steps and can create additional problems related to accuracy as each cross-section must be analyzed separately.

Furthermore, this signal processing procedure does not allow the determination of a spatial distribution of normal displacements in the whole plane of the film, and to obtain directivity properties of the waves propagating in all directions for processing exploits data u(x,t) collected only along one direction.

In order to overcome this problem we collected the full C-scan u(x,y,t) in all area around the phased array and created 3D filters which enable in one step to separate only the waves under analysis in the required frequency bandwidth. Afterward their directivity patterns and velocity maps are extracted. The technique can be described as follows: The 3D Fourier transform is performed on the collected C-scan data:

(11) $$\;U\left(f_{x},f_{y}f\right)=FFT_{3D}\left[u\left(x,y,t\right)\right]\;$$

where FFT_3D_ is the 3D Fourier transform, $f_{x}$ and $f_{y}$ are, correspondingly, the spatial frequencies along axes *x* and *y*. This data structure is in the space–frequency domain. The wavenumbers–frequency domain spectrum $U(\xi_{x},\xi_{y},f)$ is obtained by a simple transformation of axes $\xi_{x}=2\pi \cdot f_{x}$ and $\xi_{y}=2\pi \cdot f_{y}$. Then the 3D dispersion curves $U(c_{phx},c_{phy},f)$ are obtained by transformations $c_{phx}=\xi_{x}/f$ and $c_{phy}=\xi_{y}/f$.

The filtering is performed using two 3D filters. The first one filters in a frequency domain and enables selecting the desired bandwidth. The second one filters in a spatial frequency domain and enables selecting the required wave mode:

(12) $$U_{f,f_{xy}}(f_{x},f_{y},f)=U(f_{x},f_{y},f)\cdot H_{f}(f_{x},f_{y},f)\cdot H_{f_{xy}\sin}(f_{x},f_{y},f)\;$$

where $H_{f}\left(f_{x},f_{y,\;}f\right)$ is the frequency response of the band-pass filter in the frequency domain and $H_{f_{xy}\sin}\left(f_{x},f_{y,\;}f\right)$ is the frequency response of the band-pass filter in the spatial domain. The special 3D filter was developed in order to separate the selected guided wave A_0_ mode with a specific frequency and propagating in all directions from the airborne wave.

As it was shown before the frequency bandwidth of the ultrasonic array and, correspondingly, of the collected signals is limited (between 40 kHz and 44 kHz); therefore, in order to improve the signal to noise ratio the collected signals are filtered in the frequency domain with the filter the frequency response of which is given by:

(13) $$H_{f}(f_{x},f_{y},f)=\{\begin{array}{ll} F_{1}\left(f\right) & \mathrm{if}\;f_{1}-\frac{df_{F}}{2}<f<f_{1}+\frac{df_{F}}{2}\\ F_{2}\left(f\right) & \mathrm{if}\;f_{2}-\frac{df_{F}}{2}<f<f_{2}+\frac{df_{F}}{2}\\ 1 & \mathrm{if}\;f_{1}+\frac{df_{F}}{2}\leq f\leq f_{2}-\frac{df_{F}}{2}\\ 0 & \mathrm{in}\;\mathrm{other}\;\mathrm{cases} \end{array}\;$$

where $f_{1}$ and $f_{2}$ are cut-off frequencies at the −6 dB level; *df_F_* is the frequency range at the cut-off frequencies in which the frequency response of the band-pass filter smoothly reduces from 1 to 0, $F_{1}\left(f\right)=0.5\cdot \sin\left(\pi \cdot \frac{\left|f-f_{1}\right|}{df_{F}}\right)+0.5$ and $F_{2}\left(f\right)=-0.5\cdot \sin\left(\pi \cdot \frac{\left|f-f_{2}\right|}{df_{F}}\right)+0.5$ are smoothing functions for the left and right boundaries of the filter. 

The filtering in the spatial frequency domain $f_{x}0$$f_{y}$ is used in order to select the required type of wave and suppress other waves, but it is more complicated. If, to assume that ultrasonic waves propagate in the film in all directions uniformly with the same velocity *v*_w_ and the frequency *f_w_*, then the spatial filter in the $f_{x}$0$f_{y}$ plane will appear as a circle with the radius $f_{w,xy}=1/\lambda_{w}=f_{w}/c_{w}$. The selected type of the wave, for example, A_0_ mode may be filtered in the spatial frequency domain $f_{x}$0$f_{y}$ by the circular band-pass filter:

(14) $$H_{f_{xy}\sin}(f_{x},f_{y},f)=\{\begin{array}{ll} 0.5\cdot \sin\left(\pi \cdot \frac{\left|f_{xy}-f_{xy1}\right|}{df_{xyF}}\right)+0.5 & \mathrm{if}\;f_{xy1}-\frac{df_{xyF}}{2}<f_{xy}<f_{xy1}+\frac{df_{xyF}}{2}\\ -0.5\cdot \sin\left(\pi \cdot \frac{\left|f_{xy}-f_{xy2}\right|}{df_{xyF}}\right)+0.5 & \mathrm{if}\;f_{xy2}-\frac{df_{xyF}}{2}<f_{xy}<f_{xy2}+\frac{df_{xyF}}{2}\\ 1 & \mathrm{if}\;f_{xy1}+\frac{df_{xyF}}{2}\leq f_{xy}\leq f_{xy2}-\frac{df_{xyF}}{2}\\ 0 & \mathrm{in}\;\mathrm{other}\;\mathrm{cases} \end{array}\;$$

where $f_{xy}=\sqrt{f_{x}^{2}+f_{y}^{2}}$; $f_{xy1}$ and $f_{xy2}$ are cut-off spatial frequencies, and $df_{xyF}$ is the spatial frequency zone at the cut-of frequencies in which the frequency response and $H_{f_{xy}\sin}\left(f_{x},f_{y,\;}f\right)$ smoothly reduces from 1 to 0.

**Figure 19:**
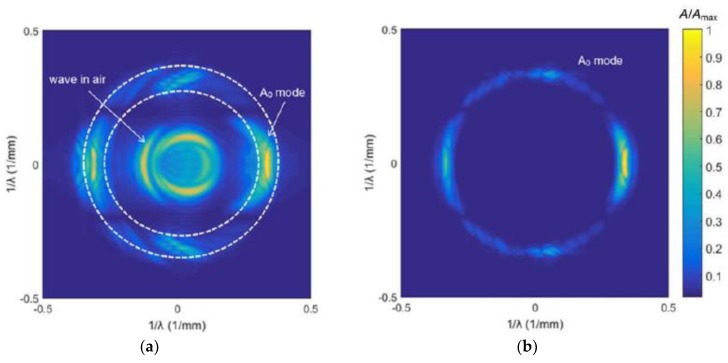

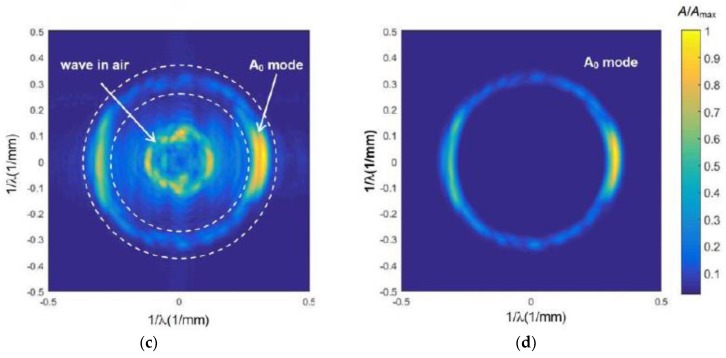
The 3D spectra of the simulated (**a**,**b**) and measured (**c**,**d**) ultrasonic fields: (**a**) before 3D filtering; (**b**) after 3D filtering; (**c**) before 3D filtering; and (**d**) after 3D filtering. The presented amplitude values shown in the color bar were normalized with respect to the maximum value in the field.

In Figure 19a,c the 3D spectra $U\left(f_{x},f_{y,\;}f\right)$ of the simulated and measured ultrasonic fields u(x,y,t) excited by the eight-element phased air-coupled array are presented. The spectra are shown in the spatial frequency domain plane as $U_{\mathrm{Cscan}}\left(f_{x},f_{y}\right)=\underset{f}{\max}{\left[\left|U\left(f_{x},f_{y},f\right)\right|\right]}^{}$. The dashed circular lines indicate cut-off spatial frequencies of the band-pass filter described by Equation (14). Figure 19b,d show the spatial spectra after the spatial filtering. In this case, the spatial cut-off frequencies were selected in order to filter away all types of waves except the A_0_ mode.

**Figure 20:**
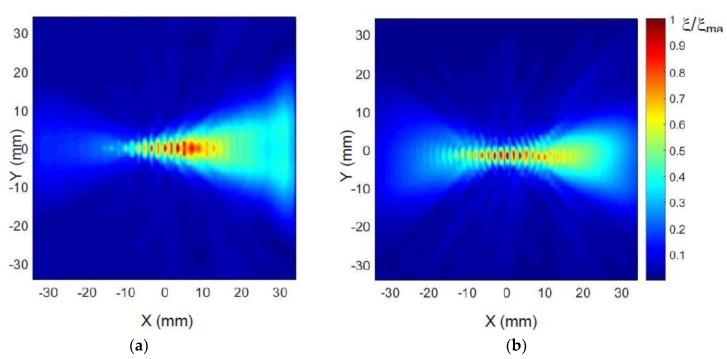
2D spatial distribution of normal displacements ξ of the plastic film after 3D filtering: (**a**) calculated; and (**b**) measured. The presented amplitude values shown in the color bar were normalized with respect to the maximum value in the field.

The spatial distributions (C-scan) of the normal displacements in the plastic film after the proposed 3D filtering are presented in Figure 20. They clearly show that with the air-coupled phased array a strong ultrasonic wave propagating to the right side from the array is excited.

**Figure 21:**
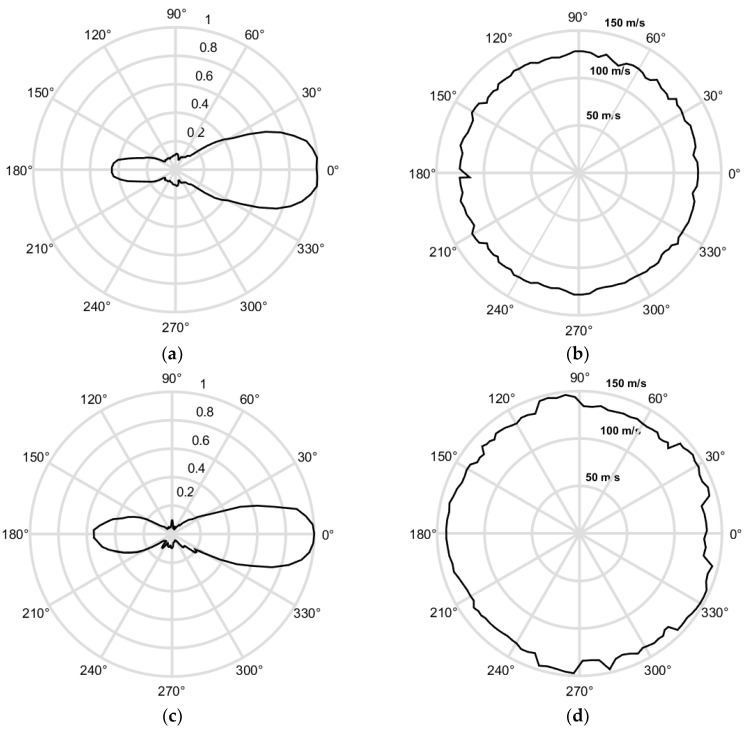
Results after 3D filtering: (**a**) simulated directivity pattern; (**b**) simulated velocity of the A_0_ mode in the film; (**c**) measured directivity pattern; and (**d**) measured velocity of the A_0_ mode in the film.

That also follows from the directivity patterns of the phased array obtained from the simulated and measured C-scans presented in Figure 15 and Figure 17. Those directivity patterns are shown in Figure 21a,c. In this figure, the normalized directivity patterns are shown:

(15) $$U_{\mathrm{DP}}^{N}(\alpha )=U_{\mathrm{DP}}(\alpha )/\max\left[U_{\mathrm{DP}}(\alpha )\right]\;$$

where the directivity pattern *U*_DP_(α) is determined as a maximum of the filtered 3D inverse Fourier transform $u_{F}\left(x,y,t\right)=FFT_{3D}^{-1}\left[U_{f,f_{xy}}\left(f_{x},f_{y},f\right)\right]$ at the distance $d=\sqrt{x_{k}^{2}+y_{l}^{2}}$ in the direction $\alpha =arctg\frac{y_{l}}{x_{k}}$:

(16) $$\;U_{DP}\left(\alpha \right)=\underset{t\in 0,T_{\max}}{\max}\left[u_{F}\left(d,\alpha ,t\right)\right]\;$$

From the results presented in Figure 21a,c follows that the proposed excitation technique with the air-coupled array allows the excitation of a low-frequency directional guided wave.

The proposed signal processing method based on the 3D Fourier transform allows also to obtain a distribution of velocities of the filtered wave propagating in different directions from the array. The velocity map can be determined using the found spectrum maximum positions in the 3D spectrum along those directions:

(17) $$\left(f_{x\max}\left(\alpha \right),f_{y\max}\left(\alpha \right)\right)=\arg\left\{\underset{\left(f_{x},f_{y}\right)\in \left[\alpha =\arctan\left(f_{y}/f_{x}\right)\right]}{\max}\left[\underset{f}{\max}\left[\left|U\left(f_{x},f_{y},f\right)\right|\right]\right]\right\}\;$$

The phase velocities of the waves propagating in different directions are found from:

(18) $$c_{\mathrm{ph}}\left(\alpha \right)=f_{\mathrm{DP}}\left(\alpha \right)\cdot \lambda_{\max}(\alpha )\;$$

where *f_DP_* (α) is the frequency corresponding to the maximum of the 3D spectrum *U*(*f_x_, f_y_*, *f*):

(19) $$f_{\mathrm{DP}}\left(\alpha \right)=\arg\left\{\underset{f}{\max}\left[\left|U\left(f_{x\max}\left(\alpha \right),f_{y\max}\left(\alpha \right),f\right)\right|\right]\right\}\;$$

and _max_(α) is given by:

(20) $$\lambda_{\max}\left(\alpha \right)=1/\sqrt{f_{x\max}^{2}\left(\alpha \right)+f_{y\max}^{2}\left(\alpha \right)}\;$$

Distributions of the velocity of the guided wave in the plastic film calculated by the proposed method using simulated and experimental data are shown in Figure 21b,d. From the obtained ultrasound velocity graphs follows that the velocity of the A_0_ mode in all directions is approximately the same, which shows that the investigated PVC film is isotropic. In the case of anisotropic materials, the velocity map would not be circular. Some random variations in the case of experimentally-obtained data are due to a lower signal to noise ratio in directions where the directivity pattern possesses lower values (Figure 21a,c). The velocity of the A_0_ mode obtained from the filtered simulated data is 125.5 m/s what is very close to the velocity of A_0_ mode *v_ph_* = 125.0 m/s at *f* = 43 kHz obtained from the dispersion curve (Figure 1). The velocity of A_0_ mode found from the filtered experimental data is *v_ph_* = 132.9 m/s. Some difference from the simulation results may be due to the fact that in simulations elastic properties of the film given by a manufacture were used. The obtained ultrasound velocity values prove that, by the proposed method, it is possible to excite a slow A_0_ Lamb wave mode and to separate it from other waves propagating in air.

## 8. Discussion and Conclusions

The performed investigation has shown that the slowA_0_ mode Lamb wave in thin plastic films may be efficiently excited by air-coupled PMN-32%PT ultrasonic arrays. Application of PMN-32%PT elements in the array allowed a significantly improved efficiency (up to five times) of the excitation in comparison to piezoelectric ceramic elements. When the velocity of the A_0_ mode in the film is less than the ultrasound velocity in air no leaky wave is observed in the surrounding air. However, propagation of the slow Lamb wave is accompanied by an evanescent wave, which exists only in close vicinity of the film and is moving together with the A_0_ mode. On the other hand, it was found that, close to the film, a wave propagates in air caused by the ultrasonic array. This airborne wave affects the film causing vibrations of the same frequency as the main A_0_ mode wave in the film. The phase of this vibration depends on the distance from the array. Those two waves interfere causing periodic amplitude variations along their propagation path. The influence of the airborne wave may be eliminated by the proposed 3D filtering in a spatial–temporal domain. After that, measurements of the parameters of the slow A_0_ mode, such as a propagation velocity in the plastic film, may be performed and applied for the material characterization. It should be noted that the velocity of the slow A_0_ Lamb wave mode in the low-frequency range strongly depends on the frequency and thickness of the film. This allows exploiting it for monitoring thickness variations of such films both off-line and on-line.

## Figures and Tables

**Table 1:** PVC material parameters.

Parameter	Value
Density	*ρ* = 1400 kg/m^3^
Young modulus	*E* = 2937 MPa
Poisson’s coefficient	*ν* = 0.42
